# Patient and carer experiences of clinical uncertainty and deterioration, in the face of limited reversibility: A comparative observational study of the AMBER care bundle

**DOI:** 10.1177/0269216315578990

**Published:** 2015-10

**Authors:** Katherine Bristowe, Irene Carey, Adrian Hopper, Susanna Shouls, Wendy Prentice, Ruth Caulkin, Irene J Higginson, Jonathan Koffman

**Affiliations:** 1 King’s College London, Cicely Saunders Institute, Department of Palliative Care, Policy and Rehabilitation, London, UK; 2 Guy’s and St Thomas’ NHS Foundation Trust, London, UK; 3 King’s College Hospital NHS Foundation Trust, London, UK

**Keywords:** Palliative care, terminal care, end-of-life care, communication, evaluation, satisfaction, hospital care, home care

## Abstract

**Background:**

Clinical uncertainty is emotionally challenging for patients and carers and creates additional pressures for those clinicians in acute hospitals. The AMBER care bundle was designed to improve care for patients identified as clinically unstable, deteriorating, with limited reversibility and at risk of dying in the next 1–2 months.

**Aim:**

To examine the experience of care supported by the AMBER care bundle compared to standard care in the context of clinical uncertainty, deterioration and limited reversibility.

**Design:**

A comparative observational mixed-methods study using semi-structured qualitative interviews and a followback survey.

**Setting/participants:**

Three large London acute tertiary National Health Service hospitals. Nineteen interviews with 23 patients and carers (10 supported by AMBER care bundle and 9 standard care). Surveys completed by next of kin of 95 deceased patients (59 AMBER care bundle and 36 standard care).

**Results:**

The AMBER care bundle was associated with increased frequency of discussions about prognosis between clinicians and patients (*χ*^2^ = 4.09, *p* = 0.04), higher awareness of their prognosis by patients (*χ*^2^ = 4.29, *p* = 0.04) and lower clarity in the information received about their condition (*χ*^2^ = 6.26, *p* = 0.04). Although the consistency and quality of communication were not different between the two groups, those supported by the AMBER care bundle described more unresolved concerns about caring for someone at home.

**Conclusion:**

Awareness of prognosis appears to be higher among patients supported by the AMBER care bundle, but in this small study this was not translated into higher quality communication, and information was judged less easy to understand. Adequately powered comparative evaluation is urgently needed.

**What is already known about the topic?**

- Clinical uncertainty towards the end of life is emotionally challenging for patients and carers and professionally challenging for clinicians.
- Poor communication is a common problem in health care, especially in the more advanced stages of disease.

**What this paper adds?**

- The AMBER care bundle may improve awareness of prognosis and frequency of discussions between clinicians and patients and carers in the presence of clinical uncertainty. The AMBER care bundle did not appear to improve patient or family reported views of communication, and those who were cared for supported by AMBER had more concerns regarding home care.

**Implications for practice, theory or policy**

- Poor communication in the acute hospital setting continues to be an issue. Robust evaluation of the AMBER care bundle is urgently needed.

## Introduction

Clinical uncertainty towards the end of life is distressing for patients and families.^1–3^ However, sharing the clinical situation, including uncertainty, is central to establishing preferences and priorities, enabling collaborative decision making^4^ and empowering patients and carers.^5–7^ While clinicians’ disclosure of clinical uncertainty with patients can be associated with increased satisfaction,^8,9^ without explanation it is associated with poor satisfaction, mistrust and loss of confidence in the clinicians.^10–13^

Recent reports have highlighted failings in open and honest communication with patients and carers.^14,15^ Care in acute hospitals often focuses on immediate clinical problems with little recognition of transitions between clinical phases^16^ and inadequate communication with patients and families.^17^ Throughout their last year of life, people spend up to 1 month in hospital; currently, 53% of all deaths in England occur in hospital,^18^ despite most people expressing a preference to be cared for, and die, at home.^19^ This has led to calls for preferences and priorities to be discussed earlier in patients’ disease trajectories.^20^

The AMBER care bundle was developed to improve care for patients in the acute hospital setting who are deteriorating, clinically unstable, with limited reversibility and at risk of dying in the next 1–2 months.^21^ This intervention has an algorithmic approach and is intended to encourage the clinical team to develop and document a clear medical plan and consider anticipated outcomes and resuscita-tion and escalation status; this is revisited daily (see Supplementary Appendix 1). The bundle also aims to increase frequency of communication with patients and family regarding treatment plans, preferred place of care and other concerns. While it prompts advance care planning, the AMBER care bundle differs from advance care planning tools because it shapes current management as well as plans for future care. The AMBER care bundle has been identified as a key enabler in the transforming end-of-life care in acute hospital programme^22^ and is now being piloted or used across 38 hospitals in England. Moreover, it is also being piloted in nine hospitals in New South Wales, Australia. Importantly, it has not yet been evaluated in a comparative study.^23^ This study, therefore, aimed to examine the experience of care supported by the AMBER care bundle compared to standard care in the context of clinical uncertainty, deterioration and limited reversibility.

## Methods

### Design

In this mixed-methods^24^ observational study, we compared the experiences of care for people supported by the AMBER care bundle with those receiving standard care, using contemporary qualitative interviews with patients and carers and followback surveys of bereaved caregivers.

### Setting

There were three large London acute tertiary National Health Service (NHS) hospitals: two where the AMBER care bundle was fully implemented (comparison wards not possible) and hospital 3 with implementation on five wards (permitting comparison). The AMBER care bundle wards in hospital 3 were as follows: one respiratory, two endocrinology, one neurology and one health and ageing. The comparison (standard care) wards were as follows: two acute medical, two health and ageing and one stroke.

### Contemporary interviews

#### Purposive sampling

We purposively sampled for heterogeneity across the two groups in order to understand how care was perceived and understood among different people with different characteristics. Potential patient participants under the care of a palliative care team were selected according to the following criteria:

- AMBER care bundle status
  - AMBER – supported by the AMBER care bundle (hospitals 1–3)
  - Comparison – would be appropriate for care supported by the AMBER care bundle if they were on an AMBER care bundle ward (hospital 3 only)
- Disease
  - Cancer
  - Non-cancer.

A family member was approached where the patient was too unwell to participate. Potential participants were not approached for the study if they lacked capacity to provide informed consent, were considered too distressed or were too unwell to participate.

#### Recruitment

Identification and first approach for the study was by the palliative care team (February–June 2013). Participants provided informed consent before commencing the interview with the researcher (K.B.), a sociolinguist with extensive experience of interviewing in palliative care research.

#### Data collection

The topic guide, shaped by a literature review, explored participants’ experiences of care and involvement in treatment decisions while in hospital, including the following:

Illness history
  - Reason for admission and recent illness
  - Patient’s main problems, symptoms and concerns
  - Whether and how the health-care team have been able to help with these concerns
Information and communication
  - Clarity of information
  - Consistency of information
  - Opportunities to ask questions
  - How patient or carer concerns were managed
  - Whether they felt that their concerns were listened to
Involvement in decision making
  - Involvement in decision about the patient’s care
  - Opportunities to talk about the future and future care
  - Unresolved concerns
Experience of care provided
  - Confidence in the care and treatment provided
  - Experiences of how different care providers had worked together.

All interviews were audio-recorded and transcribed verbatim. Recruitment continued until data saturation was achieved, and no new themes were emerging from the interviews.

#### Analysis

All interviews were analysed by K.B. using thematic analysis in five stages: familiarisation, coding, theme development, defining themes and reporting.^25^ To address issues of analytical rigour and trustworthiness, a subset of transcripts were double-coded by J.K. A re-iterant process of discussing areas of agreement and disagreement took place between K.B. and J.K. to achieve consensus. Alternative interpretations were incorporated in the analysis. The analysis was further tested during discussions with colleagues and meetings of the project advisory steering group. Attention was also paid to non-confirmatory cases where emerging themes contradicted more common ideas.^26^ Comparison was made between the AMBER care bundle and comparison groups for each emergent theme. To preserve anonymity, participants were pseudo-anonymised. Analysis was managed using NVivo qualitative analysis software (Version 10).

### Followback survey

#### Identification

Next of kin (NOK) were identified from electronic patient records (EPRs) for patients who had died within 100 days of discharge following an admission between December 2011 and December 2012. One group had received care supported by the AMBER care bundle; the other was a comparison group. Comparison patients were identified by a hospital consultant and clinical nurse specialist independently examining content of EPR data to identify patients who, all things being equal, would have been appropriate for care supported by the AMBER care bundle if they were on an AMBER care bundle ward. Criteria for selection were deterioration, clinical instability, limited reversibility and being at risk of dying within 1–2 months as available in clinical records.

#### Recruitment

All identified NOK were sent a letter from the palliative care team 4–10 months following bereavement, with the survey and a Royal College of Psychiatrists bereavement support leaflet. If no response, a second pack was sent 1 month later.

#### Date collection

We used a modified QUALYCARE postal survey,^27^ highly acceptable to participants in bereavement research.^28^ This examines the last 1–2 months of the decedent’s life, including quality and consistency of information and communication with clinicians.

#### Analysis

Analysis compared the groups, in particular questions about communication, information sharing, awareness of illness and length of stay, using independent *t*-tests and *χ*^2^ tests. Statistical significance (two-sided) was set at *p* < 0.05. For the followback survey, we calculated sample size estimates in relation to variables that were central to understanding to what extent the AMBER care bundle achieved its goals. Therefore, we wanted to detect potential differences in the percentage of respondents unable to gain sufficient information regarding a patient’s condition; distributions from followback surveys varied between 39% and 64%.^29,30^ With power set at 80% and alpha at 0.05, similarly 69 cases would be required from each group to detect this difference. In order to detect differences in the percentage of patients who knew they were going to die, previous studies have ranged between 51% and 69%.^31,32^ With power set at 80% and alpha at 0.05, we estimated a minimum of 113 cases would be required from each group to detect a similar difference.

#### Integration of data

The interview and survey data were integrated at the point of analysis, in a convergent design, interrogating the data around common key themes and questions,^33^ as demonstrated through the integrated results presented below.

## Results

### Participants

#### Contemporary interviews

A total of 23 patients and informal carers participated in 19 interviews (Table 1) – 8 interviews with patients only, 3 interviews with patient and carer dyads (patient and partner or spouse), and 8 interviews with carers only (including spouse, mother, brother, son, daughter, daughter in law, and niece), one of which was with two carers together. 10 AMBER care bundle and 9 comparison, where 11 had cancer and 8 non-cancer diagnoses (brain tumour, spinal tumour, dementia, stroke, hip fracture, heart failure, liver failure and kidney failure). Mean interview duration was 29 min (range: 11–123 min).

**Table 1.** Interview participants.

Interviews	19
Group	
AMBER care bundle	10
Comparison	9
Interview participants	
Patient only	8
Patient and carer	3
Carer(s) only	8
Patient age (years)	
40–59	6
60–79	7
80 and over	6
Mean	69
Median	70
Range	42–93
Patient gender	
Female	8
Male	11
Patient ethnicity	
Black African	1
Black Caribbean	2
White Other	3
White British	13
Disease group	
Cancer	11
Non-cancer	8
Patient deceased?	
Yes	18
No	1
<1 week after interview	6
<1 month after interview	7
<3 months after interview	3
3–6 months after interview	2
Recovered	1
Interview duration (min)	
Mean	29
Range	11–123

#### Followback survey

A total of 482 surveys were sent to the NOK: 261 deceased patients supported by the AMBER care bundle and 221 who received standard care (Table 2). Overall response rate was 20% (*n* = 95). Although the response rate differed between the two groups (AMBER care bundle 23% and comparison 16%), this was not statistically significant (*χ*^2^ = 3.01, *df* = 1, *p* = 0.08).

**Table 2.** Followback survey participants.

Mailed out to	AMBER care bundle (*n* = 261)	Comparison (*n* = 221)	Combined (*n* = 482)
Completed and returned	23% (*n* = 59)	16% (*n* = 36)	20% (*n* = 95)
Refused	9% (*n* = 24)	10% (*n* = 21)	9% (*n* = 45)
Addressee not known	13% (*n* = 34)	10% (*n* = 21)	11% (*n* = 55)
No response	55% (*n* = 144)	64% (*n* = 143)	60% (*n* = 287)
Patient characteristics	AMBER care bundle (*n* = 59)	Comparison (*n* = 36)	Combined (*n* = 95)
Age (years)			
Mean (range)	73 (28–102)	83 (61–95)	77 (28–102)
Reason for admission			
Pneumonia	20% (*n* = 12)	11% (*n* = 4)	17% (*n* = 16)
Respiratory disease (COPD, bronchiectasis and pulmonary fibrosis)	7% (*n* = 4)	14% (*n* = 5)	10% (*n* = 9)
Cancer (local and metastatic)	24% (*n* = 14)	11% (*n* = 4)	19% (*n* = 18)
Sepsis (other than pneumonia)	14% (*n* = 8)	11% (*n* = 4)	13% (*n* = 12)
MND, MS and neurodegenerative	10% (*n* = 6)	0	6% (*n* = 6)
Renal failure	3% (*n* = 2)	5% (*n* = 2)	4% (*n* = 4)
Heart failure and acute coronary syndromes	5% (*n* = 3)	11% (*n* = 4)	7% (*n* = 7)
Dementia	2% (*n* = 1)	3% (*n* = 1)	2% (*n* = 2)
Stroke and subdural haemorrhage	2% (*n* = 1)	17% (*n* = 6)	7% (*n* = 7)
Liver failure and GI disease	10% (*n* = 6)	0%	6% (*n* = 6)
Other	3% (*n* = 2)	17% (*n* = 6)	9% (*n* = 8)
Disease group			
Cancer	34% (20)	25% (9)	31% (29)
Non-cancer	66% (39)	75% (27)	69% (66)
Gender			
Male	46% (*n* = 27)	53% (*n* = 19)	48% (*n* = 46)
Female	54% (*n* = 32)	47% (*n* = 17)	52% (*n* = 49)
Ethnicity			
White British/Other	85% (*n* = 50)	72% (*n* = 26)	80% (*n* = 76)
Black African/Caribbean/Other	5% (*n* = 3)	14% (*n* = 5)	9% (*n* = 8)
Asian	5% (*n* = 3)	3% (*n* = 1)	4% (*n* = 4)
Other	2% (*n* = 1)	8% (*n* = 3)	4% (*n* = 4)
Not completed	3% (*n* = 2)	3% (*n* = 1)	3% (*n* = 3)
Next of kin or respondent characteristics			
Gender			
Male	24% (*n* = 14)	31% (*n* = 11)	26% (*n* = 25)
Female	76% (*n* = 45)	67% (*n* = 24)	73% (*n* = 69)
Not completed		2% (*n* = 1)	1% (*n* = 1)
Age (years)			
Mean	60 (*n* = 58)	65 (*n* = 32)	62 (*n* = 90)
Range	21–87	44–91	21–91
Not completed	1	4	5
Ethnicity			
White British/Other	89% (*n* = 52)	72% (26)	82% (*n* = 78)
Black African/Caribbean/Other	5% (*n* = 3)	13% (5)	9% (*n* = 8)
Asian	0% (*n* = 0)	3% (1)	1% (*n* = 1)
Other	3% (*n* = 2)	6% (2)	4% (*n* = 4)
Not completed	3% (*n* = 2)	6% (2)	4% (*n* = 4)

COPD: chronic obstructive pulmonary disease; MND: motor neuron disease; MS: multiple sclerosis; GI: gastrointestinal.

The first section of the results will present the differences between the AMBER care bundle and standard care experiences for patients and carers, and the second section will present the similarities.

1. *Exploring the AMBER care bundle and comparison group experiences (survey and interviews) – differences*

In both the interviews and survey, differences emerged in the experiences of those in the AMBER care bundle and comparison groups. Specifically, these were related to awareness of the clinical situation and discussion and realisation of preferences for place of care. For each of these, the experiences shared in the followback survey and contemporary interviews are described below.

### Awareness of the clinical situation

#### Followback survey

The AMBER care bundle family meeting is an opportunity to discuss prognosis, preferences, priorities and concerns. Significantly more of the AMBER care bundle group than the comparison group reported that the patient was aware they were going to die from their illness (72% compared to 48%, *χ*^2^ = 4.29, *p* = 0.04; Table 3). Also, significantly more of the AMBER care bundle group recalled a clinician discussing with the patient that they were likely to die from their illness (59% compared to 32%, *χ*^2^ = 4.09, *p* = 0.04; Table 3).

**Table 3.** Followback survey results.

Awareness of prognosis			
	AMBER care bundle (*n* = 53)	Comparison (*n* = 27)	*p* value
Was the patient aware they were going to die because of their illness?			
Yes, certainly or probably knew	38 (72%)	13 (48%)	*χ*^2^ = 4.29, *df* = 1, *p* = 0.04
No, probably or definitely did not know	15 (28%)	14 (52%)	
	AMBER care bundle (*n* = 41)	Comparison (*n* = 22)	*p* value
Did any health professional discuss with the patient that he or she was likely to die from the illness?			
Yes	24 (59%)	7 (32%)	*χ*^2^ = 4.09, *df* = 1, *p* = 0.04
No	17 (41%)	15 (68%)	
Length of hospital stay (days)			
	AMBER care bundle (*n* = 41)	Comparison (*n* = 19)	*p* value
Length of hospital stay for all patients	Mean: 20.3 (SD: 19.2, median: 14, range: 1–87)	Mean: 29.3 (SD: 20.4, median: 21, range: 6–70)	*t*-test = −1.65, *df* = 58, *p* = 0.10
	AMBER care bundle (*n* = 20)	Comparison (*n* = 9)	*p* value
Length of hospital stay for all patients who were discharged and died in a place *other than* hospital	Mean: 17.6 (SD: 14.6, median: 13.5, range: 1–87)	Mean: 21.4 (SD: 15.1, median: 14, range: 6–70)	*t*-test = −0.66, *df* = 27, *p* = 0.52
Communication and information sharing			
	AMBER care bundle (*n* = 57)	Comparison (*n* = 35)	*p* value
Did you receive information about his condition that was clear and easy to understand?			
Yes, most of the time	29 (51%)	24 (69%)	*χ*^2^ = 6.26, *df* = 2, *p* = 0.04
Sometimes	16 (28%)	10 (28%)	
No, not at all	12 (21%)	1 (3%)	
	AMBER care bundle (*n* = 55)	Comparison (*n* = 34)	*p* value
Do you remember receiving information on a day-to-day basis that helped you understand the reason for the care he or she received?			
Yes, most of the time	21 (38%)	17 (50%)	*χ*^2^ = 1.54, *df* = 2, *p* = 0.46
Sometimes	19 (35%)	11 (32%)	
No, not at all	15 (27%)	6 (18%)	
	AMBER care bundle (*n* = 55)	Comparison (*n* = 35)	*p* value
Did you receive consistent information about his condition?			
Yes, most of the time	25 (45%)	18 (52%)	*χ*^2^ = 2.75, *df* = 2, *p* = 0.25
Sometimes	14 (26%)	12 (34%)	
No, not at all did not know	16 (29%)	5 (14%)	
Involvement of palliative care			
	AMBER care bundle (*n* = 48)	Comparison (*n* = 28)	
Was the patients seen by someone from the palliative care team or the Macmillan nurses at the hospital?	29 (60%)	17 (61%)	
Place of death			
	AMBER care bundle (*n* = 51)	Comparison (*n* = 28)	*p* value
As far as you know, where would the patient have preferred to die?			
Home or home of relative or close friend	45% (*n* = 23)	39% (*n* = 11)	*χ*^2^ = 3.92, *df* = 4, *p* = 0.42
Hospice	24% (*n* = 12)	14% (*n* = 4)	
Hospital	21% (*n* = 11)	36% (*n* = 10)	
Nursing or residential care home	6% (*n* = 3)	11% (*n* = 3)	
Elsewhere	4% (*n* = 2)	0% (*n* = 0)	
	AMBER care bundle (*n* = 59)	Comparison (*n* = 35)	*p* value
Where did the patient die?			
Home or home of relative or close friend	20% (*n* = 12)	9% (*n* = 3)	*χ*^2^ = 5.71, *df* = 3, *p* = 0.13
Hospice	20% (*n* = 12)	9% (*n* = 3)	
Hospital	51% (30)	68% (24)	
Nursing or residential care home	9% (*n* = 5)	14% (*n* = 5)	

SD: standard deviation.

#### Contemporary interviews

Carers supported by the AMBER care bundle described clinicians deliberately seeking them out to update them and address concerns, which represented a source of enormous support:

one of the doctors actually rung me from home at nine o clock at night once because she realised she’d forgotten or hadn’t had a chance to come and see me so that was … was really nice and that was much appreciated. (Cheryl, daughter of a man with lung cancer – AMBER)

Further differences were evident in the contemporary interviews. Although both groups reported difficulties with inconsistent information, for those supported by the AMBER care bundle, incomplete or inconsistent information was often described in the context of rapidly changing clinical situations, as a cause of clinical uncertainty, as illustrated below (emphasis added):

As I say it’s just … not knowing exactly like, that’s the only thing that bothers me … *they can’t predict* … but I know he is getting weaker. (Mary, wife of man with heart failure – AMBER)

Contrastingly, participants in the comparison interviews tended to report uncertainty as a result of the clinicians’ ‘assumptions’, due to their inadequate or incomplete knowledge, suggesting that perhaps the clinical uncertainty had not been fully explained (emphasis added):

They come in everyday … the only question I’ve got and they can’t answer is exactly how long have I got … *they don’t know … it’s all assumption*. (Martin, man with bladder cancer – Comparison)

These differences suggest those supported by the AMBER care bundle may have more of an understanding of the clinical uncertainty and its resultant impact upon clinicians’ ability to provide consistent and complete information.

### Discussion and realisation of preferences for place of care

#### Followback survey

Length of hospital stay was not significantly different between the groups: AMBER care bundle group mean hospital stay 20.3 days (range: 1–87 days) and comparison group 29.3 days (range: 6–70 days; Table 3). Broadly, similar proportions of patients cared for on AMBER care bundle wards (45%, *n* = 23/51) and the comparison group (39%, *n* = 11/28) were known to have preferred a home death. However, the numbers of AMBER (20%, *n* = 12/59) and comparison group (8%, *n* = 3/35) patients who did so (or the home of a relative or friend) differed, although this difference was not statistically significant.

#### Contemporary interviews

Concerns of caring for a relative at home were more evident among those supported by the AMBER care bundle than the comparison group, including feeling unprepared practically and emotionally for the experience, questioning whether they would cope and facing the physical reality of death of a relative at home. Although preferred place of care was elicited and potentially expedited, concerns regarding discharge were not addressed drawing into question the quality of the discussions:

Even now we’re still having conversations about … are you going to go home and … although he wants to I don’t think that it’s perhaps the best place and I don’t think that he really thinks that it’s the best place … from a practical point of view … so I guess maybe you know if someone had said … actually … have you really thought about the implications of this … might have been useful … um at the time when he was saying yes I want to go home. (Cheryl, daughter of a man with stomach cancer – AMBER)

These unresolved concerns were also found in the followback survey free-text entries (Table 4).

**Table 4.** Followback survey free-text comments.

Preferred place of care		
AMBER/comparison	Respondent	Comment
AMBER	Son of woman with cancer	I felt hopeless and worried at times when my mum complained about pain. I didn’t know how to help her, especially when she was in a coma and sweating a lot. At the time I wished she was in a hospice.
AMBER	Daughter of woman with cancer	My mum was sent to a rehabilitation centre because she couldn’t look after herself at home. She was too ill for rehabilitation but the hospice did not seem to be an option. Had I known she didn’t have long to live, I would have made sure she stayed at home and I would have looked after her.
Communication		
AMBER or comparison	Respondent	Comment
AMBER	Daughter of woman with cancer	Doctors were pretty poor at making time to explain things. Poor communication between them meant hard to know where we stood, next steps etc.
AMBER	Daughter of woman with COPD	Doctors took time to speak to me and explain what was wrong and what they were doing to help.
AMBER	Daughter of woman with cancer	It was often quite difficult to get information about her condition and what treatment was being given. Doctors rarely available to talk to at the times I was able to visit, and nurses were not able to discuss her case.
AMBER	Son of woman with sepsis	We the family were treated with kindness and kept fully informed.
AMBER	Daughter of woman with cancer	I would have appreciated knowing earlier that she was coming to the end of her life. I think I knew but I needed to know on admission that she would not survive. I needed someone to tell me, even though the staff may have thought I knew, I needed confirmation to act accordingly.
Comparison	Relative of woman with sepsis	They explained what they thought was wrong with her to her family but when death was near, never told the family that she was close to the end of life.
Comparison	Wife of man who had a stroke	Quite good communication when doctors on ward, but it was very difficult to find out information on a day to day basis as family went days without seeing a doctor.
Comparison	Wife of man who had a stroke	I am not sure they explained adequately the situation. He could not speak but his brain was intact.
Comparison	Husband of woman with subdural haemorrhage	Care was taken, but information was never explained to me.

COPD: chronic obstructive pulmonary disease.

2. *Communication concerns (survey and interviews) – similarities*

There were also some similarities to the experiences shared by the two groups. Both those supported by the AMBER care bundle and the comparison group described challenges when communicating with clinicians. Concerns were related to the actual information shared and the process of information sharing. For each of these, the experiences shared in the followback survey and contemporary interviews are described below.

### Information shared

#### Followback survey

Respondents from the AMBER care bundle group were less likely than the comparison group to report that information was clear and easy to understand (69% vs 51%, *χ* = 6.26, *p* = 0.04). However, there were no differences in frequency or consistency of communication with clinicians between the groups (Table 3).

#### Contemporary interviews

The impact of inconsistent information was also described by interview participants, often in very emotive terms:

We were told on a Sunday evening at seven … that … we need to make preparations to get John either to our local hospital … or indeed home because there’s nothing more that can be done for him … and then on the Monday morning to arrive and be told by his consultant that … John’s doing remarkably well … and there’s no reason that within a couple of months he shouldn’t be back up and on his feet … the emotional trauma for you … for all of us. (Tom, brother of man with spinal tumour – Comparison)

### Process of information sharing

Concerns were also raised, regarding the process of information sharing with the clinicians.

#### Followback survey

Respondents shared positive experiences of communication with clinicians but also feelings of abandonment at evenings and weekends. Few doctors were present to talk to, as described in the survey free-text entries (Table 4).

#### Contemporary interviews

Participants described the need for a single point of contact to provide continuity and avoid unnecessary confusion. Participants also described insensitivity in the manner information was shared. Several evidently distressing experiences were described in both groups:

The doctor told me we are in a situation of diminishing returns and ought to let nature take its course … this was so blunt … I couldn’t sleep for two days. (Devan, man with lung cancer – AMBER)

## Discussion

This first comparative evaluation of the AMBER care bundle found that it may be associated with increased frequency of discussions about prognosis with patients and families and improved awareness of the clinical situation. However, there were no significant differences in length of hospital stay, satisfaction with communication, or frequency and clarity of information shared.

The AMBER care bundle appeared to enable more of an understanding of the reasons underlying clinical uncertainty, increased awareness of the clinical situation and increased frequency of discussions about prognosis. However, clarity of information sharing was worse among the AMBER care bundle group than the comparison group. The use of other approaches such as proactive elderly advance care planning tools on comparison wards may have affected this. It is possible that while discussions took place among the AMBER care bundle group, the information was not communicated effectively due to lack of skills in communicating the complexity of clinical uncertainty and prognostication or increased complexity of discussions among this group with more information being shared, resulting in possible confusion. Inadequate explanation of clinical uncertainty has been found to negatively affect patient and carer experiences,^13^ and poor communication can be detrimental to patient experiences and understanding of prognosis, results and treatment plans.^34^ The findings from this study suggest that without appropriate support and training, alongside the AMBER care bundle, communication of information may remain unsatisfactory.

The AMBER care bundle appeared to enable more of an understanding of the reasons underlying clinical uncertainty, increased awareness of the clinical situation and increased frequency of discussions about prognosis. However, clarity of information sharing was worse among the AMBER care bundle group than the comparison group. The use of other approaches such as proactive elderly advance care planning tools on comparison wards may have affected this. It is possible that while discussions took place among the AMBER care bundle group, the information was not communicated effectively due to lack of skills in communicating the complexity of clinical uncertainty and prognostication or increased complexity of discussions among this group with more information being shared, resulting in possible confusion. Inadequate explanation of clinical uncertainty has been found to negatively affect patient and carer experiences,^13^ and poor communication can be detrimental to patient experiences and understanding of prognosis, results and treatment plans.^34^ The findings from this study suggest that without appropriate support and training, alongside the AMBER care bundle, communication of information may remain unsatisfactory.

Those supported by the AMBER care bundle also volunteered more concerns about the reality of ‘going home’ than the comparison group. While discussions regarding preferred place of care had occurred, there were many unresolved concerns around leaving the security of the hospital, how they would cope at home and the support that would be available. One potential criticism of the AMBER care bundle is the lack of emphasis on exploring patient and family information preferences before initiating discussions. Clinicians are poor at estimating the information and decision making preferences of patients and carers.^35–37^ Also, patient and carer preferences often differ, and carers are poor at predicting the information preferences of patients.^38^ Further communication training should accompany the AMBER care bundle, particularly in light of recent criticism of communication in the acute hospital setting,^14,15^ to ensure discussions are individually tailored accommodating patients’ and carers’ preferences for information and discussions.

### Strengths and limitations

This study represents the first attempt to evaluate the AMBER care bundle in the acute hospital setting and has important strengths. The integration of the qualitative and quantitative data enabled the researchers to ask intersecting questions, facilitating a more complete exploration of care supported by the AMBER care bundle. Also, this study involved meaningful engagement with a hard-to-reach population who were clinically unstable, deteriorating, with limited reversibility and at risk of dying in the next 1–2 months. Over half of the patients interviewed died within 1 month of participating, and many within days, providing invaluable insights into experiences at such a challenging time.

However, the study has limitations. First, the design was observational. Although there was a comparative element, the comparison and AMBER care bundle groups were likely to be different in ways other than the intervention. Second, while this study was primarily exploratory, based on our sample size calculations, we failed to recruit sufficient numbers to detect other potential important differences – the response rate being considered as a marker of success. The EPR data from which we identified the participants for the followback survey were sometimes of poor quality; some addresses were redundant or the NOK was deceased making it impossible to identify an appropriate proxy. This was a particular problem for the comparison group, which had a very poor response rate. This not only raised general concerns for hospitals about the veracity of their data but also reduced the potential number of respondents and will also have biased the comparison group.

Third, the followback survey relies on ‘proxy’ experiences of bereaved caregivers rather than patient-centred accounts. While the validity of proxy accounts has been questioned,^39,40^ this approach is often employed^27,32,41^ to overcome difficulties of obtaining views of representative samples of patients. Many studies relying on patients’ accounts prior to death are potentially biased since they represent only a small proportion of patients with an identifiable terminal illness, who are relatively well and therefore able to participate, and are willing to take part. Fourth, the choice and matching of comparison wards were challenging, and it was not possible to control for other interventions which may have altered care. In particular, many patients were supported by a palliative care team, and this may have influenced the care provided.

## Conclusion

This study found some potential benefits to care supported by the AMBER care bundle, in particular in terms of levels of knowledge of patients and caregivers. However, it also identified potential downsides, specifically concerning information and communication including about going home. This highlights the importance of ensuring adequate training when implementing the AMBER care bundle. Our study was small and may have been inadequately powered to detect other differences. Our data point to a need for robust sufficiently powered comparative evaluation of the AMBER care bundle and other similar tools and complex interventions utilised towards the end of life, including of potential benefits and harms. The findings also suggest that additional communication training is vital, as both groups in this study reported negative experiences.

## Supplementary Material

Supplementary material
