# Supplementary material for: Patient and carer experiences of clinical uncertainty and deterioration, in the face of limited reversibility: A comparative observational study of the AMBER care bundle
Source: Palliat Med. 2015 Oct;29(9):797–807. doi: 10.1177/0269216315578990 (PMC4572938; doi:10.1177/0269216315578990)
Supplement: Supplementary material [file PMJ578990_Online_Appendix.pdf]

APPENDIX 1: the AMBER care bundle

Background

The AMBER care bundle provides a systematic approach to managing the care of hospital patients who are facing an uncertain recovery and who are at risk of dying in the next 1-2 months - but not clearly in the last few days of life - and for whom active medical management may still be appropriate. It is an intervention that can fit within any care pathway or diagnostic group for patients whose recovery is uncertain.

Stage 1: Identification

Any member of the clinical multi-disciplinary team is encouraged to identify patients suitable for the AMBER care bundle using the two questions below.

Is the patient suitable for the AMBER care bundle?

1. Is the patient deteriorating, clinically unstable, and with limited reversibility; and,
2. Is the patient at risk of dying within the next 1-2 months?

Remember to apply the principles of the Mental Capacity Act 2005

Stage 2: Day One Interventions

If the answer is "Yes" to both questions then the multi-disciplinary team is expected to proceed to the four interventions in the care bundle. As the AMBER care bundle is a multi-disciplinary tool, it involves doctors, nurses and the whole team working together.

	**Intervention**<br>Assess patient capacity for each decision and involve in line with the Mental Capacity Act 2005	**Action**	**Comments**	**Name**<br>Date & Time
**Action within 4 hrs**<br>Medical responsibility to ensure intervention takes place	**Medical plan documented in patient record**<br>Including:<br>• current key issues<br>• anticipated outcomes<br>• resuscitation status	☐ Yes<br>☐ No		
	**Escalation decision documented**<br>Including:<br>☐ Ward only ☐ HDU only ☐ ITU	☐ Yes<br>☐ No		
	**Medical plan discussed and agreed with nursing staff**	☐ Yes<br>☐ No		
**Action within 12 hrs**<br>Nursing responsibility to ensure intervention takes place	**Patient ± carer discussion or meeting held and clearly documented**<br>Which may include:<br>• uncertain recovery and treatment options<br>• preferred place of care<br>• any concerns or wishes<br>• who was present	☐ Yes<br>☐ No		
**Record details in the patient's record**				

The AMBER care bundle has clear timescales for action. Patients should be involved in decision making throughout, at their own pace and in line with their assessed mental capacity. The timescales for decision making (four hours and 12 hours) were included as a result of feedback from staff as this clear timescale makes it more likely that actions and decisions are completed.

The care bundle includes decisions about cardiopulmonary resuscitation and escalation of care to critical care, but does not exclude these treatments. Some patients on the AMBER care bundle may be both for cardiopulmonary resuscitation and full escalation of treatment.

Some patients respond to active treatment and recover, whilst others do not and high quality palliative/end of life care may be appropriate. Therefore, once a patient is identified as suitable for the AMBER care bundle, they will continue to be suitable until they either recover, or they die. If a patient is discharged from hospital, their status (recovery uncertain) and relevant information including information on patient’s preferences should be communicated to relevant members of the community team.

Stage 3: Daily Monitoring and Review

When a patient's care is supported with the AMBER care bundle, both they and their carers (as appropriate) should expect to receive daily contact by staff who assess and "**ACT**". This is the point at which staff checks to see if the patient’s preferences for care have changed.

Don’t forget to ‘ACT’ daily:

Assess patient capacity for each decision and involve in line with the Mental Capacity Act 2005

A Is your patient still ‘**A**MBER’?

C Are there any medical **C**hanges?

T Have you **T**ouched base with the patient ± carer?

Review patient’s preferred place of care. Has it changed?

NB. The AMBER care bundle, described above (version 3, 2012), is currently undergoing further modification.
